# Fewer blue lakes and more murky lakes across the continental U.S.: Implications for planktonic food webs

**DOI:** 10.1002/lno.10967

**Published:** 2018-11

**Authors:** Dina M. Leech, Amina I. Pollard, Stephanie G. Labou, Stephanie E. Hampton

**Affiliations:** 1 Department of Biological and Environmental Sciences, Longwood University, Farmville, Virginia; 2 US EPA Office of Water, Washington, District of Columbia; 3 Center for Environmental Research, Education and Outreach, Washington State University, Pullman, Washington

## Abstract

Elevated allochthonous inputs of organic matter are increasingly recognized as a driver of ecosystem change in lakes, particularly when concurrent with eutrophication. Evaluation of lakes in a nutrient-color paradigm (i.e., based on total phosphorus and true color) enables a more robust approach to research and management. To assess temporal and spatial patterns in nutrient-color status for U.S. lakes and associated food web attributes, we analyzed the U.S. Environmental Protection Agency’s National Lakes Assessment (NLA) data. With 1000+ lakes sampled in 2007 and 2012 in a stratified random sampling design, the NLA enables rigorous assessment of lake condition across the continental U.S. We demonstrate that many U.S. lakes are simultaneously experiencing eutrophication and brownification to produce an abundance of “murky” lakes. Overall, “blue” lakes decreased by ~ 18% (46% of lakes in 2007 to 28% in 2012) while “murky” lakes increased by almost 12% (24% of lakes in 2007 to 35.4% in 2012). No statistical differences were observed in the proportions of “green” or “brown” lakes. Regionally, murky lakes significantly increased in the Northern Appalachian, Southern Plains, and Xeric ecoregions. Murky lakes exhibited the highest epilimnetic chlorophyll *a* concentrations, cyanobacterial densities, and microcystin concentrations. Total zooplankton biomass was also highest in murky lakes, primarily due to increased rotifer and copepod biomass. However, zooplankton : phytoplankton biomass ratios were low, suggesting reduced energy transfer to higher trophic levels. These results emphasize that many lakes in the U.S. are simultaneously “greening” and “browning”, with potentially negative consequences for water quality and food web structure.

Lakes are most often classified by their autotrophic status, or quantity of algal biomass, ranging from oligotrophic to hypereutrophic. This dominant classification scheme is likely driven by the global prevalence of eutrophication, or the “greening” of lakes, since the 1950s. Excessive algal biomass often results in reductions in water quality (Heisler et al. 2008; Smith and Schindler 2009) as well as food web stability (Jeppesen et al. 2000; Kuiper et al. 2015). Substantial efforts have therefore been made to reduce nutrient inputs to fresh waters (Jeppesen et al. 2005;Conley et al. 2009). However, eutrophication continues to be a systemic problem. A recent study indicated that the proportion of oligotrophic lakes in the continental U.S. decreased from 25% to 7% from 2007 to 2012, based on data from the U.S. Environmental Protection Agency’s aquatic resources survey (Stoddard et al. 2016). This trend was based on a comparison of lake total phosphorus (TP) concentrations, and the authors caution if TP concentrations continue to rise, oligotrophic lakes may soon be a rare feature on the North American landscape (Stoddard et al. 2016).

Many lakes in the northern hemisphere have also shown signs of “browning” due to the increased runoff of chromophoric (i.e., colored) dissolved organic matter (CDOM) (Solomon et al. 2015), and in some cases dissolved iron (Kritzberg and Ekström 2012), from the terrestrial watershed. The mechanisms driving brownification are debated, including changes in climate, hydrology, land use, and atmospheric sulfur deposition (Freeman et al. 2001; Monteith et al. 2007; Erlandsson et al. 2008; Haaland et al. 2010). Like eutrophication, as lakes increase in brown color, numerous complex changes occur to the physical and chemical environments that affect water quality and food web structure. For example, increased browning of oligotrophic lakes stimulates primary production through the addition of nutrients (Ask et al. 2012; Seekell et al. 2015; Solomon et al. 2015). However, excessive browning can reduce habitat quality for aquatic organisms, such as reductions in light for phytoplankton and visual predators, lower dissolved oxygen concentrations, and a shallowing of the mixing depth (Thrane et al. 2014; Solomon et al. 2015; Finstad et al. 2014). Lakes often shift from net autotrophic to net heterotrophic as increased inputs of organic matter resources stimulate increased bacterial decomposition, making them a source of CO_2_ to the atmosphere and contributor to global climate change (Cole et al. 1994; Larsen et al. 2011; Ask et al. 2012). In addition, lake “browning” is a human health concern as it increases the mobilization of heavy metals, such as mercury (Dittman et al. 2009), and the formation of disinfection byproducts during drinking water purification (Chow et al. 2007).

Given the possibility for lakes to “green,” “brown,” or both simultaneously, the nutrient-color paradigm represents a more appropriate classification scheme, relative to considering nutrient status alone (Williamson et al. 1999). The paradigm emphasizes both autotrophic and heterotrophic properties, identifying lakes as oligotrophic (blue), eutrophic (green), dystrophic (brown), or mixotrophic (murky) based on a lake’s total phosphorus concentration and true color. Nürnberg and Shaw (1998) and Webster et al. (2008) provide strong empirical support for the nutrient-color paradigm, noting that the effect of water color on chlorophyll *a* (Chl *a*) concentrations and Secchi depth is often independent of TP. However, these studies primarily focused on north-temperate lakes in North America. Here, we expand on this work to examine patterns in lake nutrient-color status across the conterminous U.S. (26°N–49°N) and the consequent effects on water quality and food web structure.

The fact that grazer communities often shift with algal production (Dodson et al. 2000; Jeppesen et al. 2000; Duggan et al. 2002; Pinto-Coelho et al. 2005) and that zooplankton taxa respond differentially to dissolved organic substances (Wehr et al. 1998; Cooke et al. 2015) suggests that grazer community structure likely differs with lake nutrient-color status. Some grazers specialize on only bacteria (Pourriot 1977) while others may be mechanically unable to eat bacteria but thrive on bacterivorous flagellates and ciliates (Arndt 1993). Grazers that readily consume both bacteria and algae may have a competitive advantage given the ability to switch between these two nutritional sources (Sanders et al. 1989; Berggren et al. 2014). In this study, we focus on zooplankton as they represent a central role in the aquatic food web, controlling algal abundance as well as serving as prey for higher trophic levels.

Data from the U.S. Environmental Protection Agency’s National Lake Assessment (NLA) are used to first examine the extent and magnitude of nutrient-color change in lakes between the 2007 and 2012 NLA surveys (U.S. EPA 2010, 2016). We then use data from the 2012 NLA survey to characterize differences in basal resources and zooplankton communities within the four lake types of the nutrient-color paradigm. Given the prevalence of eutrophication and brownification, we hypothesize that “blue” lakes will decrease in abundance while “green,” “brown,” and especially “murky” lakes will increase. These patterns are explored across the 48 conterminous U.S. as well as within specific ecoregions. We further hypothesize that basal pelagic resources will vary with lake nutrient-color status, and as a consequence, influence zooplankton biomass and community structure. Finally, we discuss the potential implications of changes in lake nutrient-color status for water quality and food web dynamics.

## Methods

### NLA dataset

The NLA is a synoptic sampling program of lakes, reservoirs, and ponds implemented across the conterminous U.S. on a 5-yr cycle. Approximately 1000 lakes are sampled in the summer (June–September) during each cycle. Lakes are selected from the National Hydrography Database (version 2; https://nhd.usgs.gov/) using a stratified randomized statistical design that stratifies based on aggregated Omernik level-III ecoregion and lake size. Natural and man-made lakes are treated equally in site selection, sampling protocols, and laboratory analyses’ methodology. From the statistical design, a total of 1028 lakes were sampled in the 2007 survey and 1038 lakes in the 2012 survey. Of these, 401 lakes were sampled in both assessment years. The 2007 NLA survey included lakes ≥4 ha in size while the 2012 NLA survey included lakes ≥1 ha. An extensive set of environmental variables was measured at each sampled lake, but we provide sampling details only for variables used in our analysis. All data are freely available online (https://www.epa.gov/national-aquatic-resource-surveys).

### Field sample collections

Field sampling occurred throughout the summer, from June through September, with the average sample date ~ 2 weeks earlier in 2012 than in 2007 (i.e., average sampling date was July 17 in 2012 and July 31 in 2007; paired *t*-test *p* < 0.001). Each lake was sampled only once during each survey. Field crews use standardized sampling methods across all sites, with collections made during the morning to early afternoon. Biological and chemical samples used in this analysis were collected at a deep, open water location in each lake (i.e., ≥50 m in natural lakes and at a midpoint in reservoirs). At this location, water was collected from the photic zone of the lake (i.e., ≥50 m in lakes and at the midpoint of reservoirs) with a vertical, depth-integrated method, using a 2 m PVC tube with a 3.2 cm diameter fitted with a stopper and a valve (design by the Minnesota Pollution Control Agency;U.S. EPA 2012a, Section 5.5). Water was transferred from the integrated sampling device to a triple rinsed 4 L cubitainer. Once full, the cubitainer was inverted several times to gently mix the water sample, and then, nutrient, phytoplankton, and Chl *a* subsamples were taken. The cubitainer was then filled again following the same procedure to collect subsamples for general water chemistry parameters, including true color, pH, and acidneutralizing capacity.

### Water quality data

Water samples for chemical analysis were placed on ice and shipped overnight to the Willamette Research Station in Corvallis, Oregon. All variables of interest were quantified at pre-specified levels of precision and accuracy, and methods were the same in both years (U.S. EPA 2009, 2012a). Detailed descriptions of all water quality analyses are found in the NLA 2012 Laboratory Operations Manual (Section 9, U.S. EPA 2012a). Briefly, (1) Chl *a* concentrations were measured on a fluorometer after extraction in 90% acetone, (2) nitrate-nitrite, ammonia, total nitrogen, and total phosphorus concentrations were measured with flow injection automated colorimetric analysis following sample digestion, (3) true color was estimated by visual comparison of filtered water samples to a calibrated glass color disk, (4) dissolved organic carbon (DOC) concentrations were measured using UV promoted persulfate oxidation to CO_2_ with infrared detection, (5) pH was measured with a ManSci PC-Titrate w/Titra-Sip autotitrator and Ross combination pH electrode, and (6) acid-neutralizing capacity was measured with automated acidimetric titration to pH < 3.5, with modified Gran plot analysis.

### Phytoplankton data

After collection, phytoplankton samples were immediately preserved with Lugol’s and stored on ice in the dark until shipped to BSA Environmental Services in Beachwood, Ohio. Cells were identified, measured, and enumerated in Utermöhl sedimentation chambers under a compound microscope (U.S. EPA 2012b, Section 5.4). Phytoplankton abundance (cells mL^−1^) was estimated from the count data and the volume of water sampled. Cell dimension measurements in combination with phytoplankton abundance were then used to estimate the biovolume (*μ*m^3^ mL^−1^) of each major taxonomic group.

During data processing, we removed phytoplankton orders constituting < 5% of any sample from the dataset. Of the remaining orders, genera contributing < 5% of any sample biovolume were removed. The subsequent biovolume data were converted to biomass (*μ*g L^−1^ dry weight, to match zooplankton data), assuming a 20% conversion of *μ*g L^−1^ wet weight to dry weight or an overall conversion factor of 10 mg wet weight : mg carbon (Strickland 1966). Phytoplankton biomass data were then used to compute zooplankton : phytoplankton biomass ratios in each lake. Only 2012 NLA phytoplankton data were used in our analysis because we were limited to using only 2012 NLA zooplankton data, as discussed below in “Zooplankton data”.

Given their potential negative effects on water quality and food web dynamics, particular attention was given to cyanobacterial densities as well as the concentration of microcystin across the four lake types. The latter was measured in unfiltered water samples using the Microtiter Plate Enzyme-Linked Immuno-Sorbent Assay (U.S. EPA 2012b, Section 3). Samples completed three freeze–thaw cycles to lyse cells before being processed using immunoassay kits purchased from Abraxis (Warminster, PA). Microcystin concentrations are considered potentially toxic to human health above 1 *μ*g L^−1^ in finished drinking water and above 10 *μ*g L^−1^ in recreational waters (U.S. EPA 2012b, Section 3).

### Zooplankton data

We focused on zooplankton data collected during 2012 NLA because sampling protocols differed between 2007 and 2012. In 2012, field crews used a fine (50 *μ*m) and a coarse (150 *μ*m) Wisconsin mesh nets to collect zooplankton during the day. Both nets were towed vertically a total length of 5 m to sample the same volume in each lake. However, the number of tows varied with site depth (U.S. EPA 2012a, Section 5.5). All zooplankton samples were immediately narcotized and preserved with 95% ethanol in the field. Samples collected in Wisconsin were shipped to a state laboratory while all other samples were shipped to BSA Environmental Services. Both laboratories used standardized protocols to identify and enumerate zooplankton, which are detailed in the NLA Laboratory Operations Manual (U.S. EPA 2012b, Section 10.4).

Briefly, zooplankton were identified to the lowest level of taxonomic resolution, usually species. Identification, enumeration, and body measurements were performed under either a compound microscope (i.e., microzooplankton—rotifers and copepod nauplii) or a dissecting microscope (i.e., macrozooplankton) in a Sedgwick-Rafter cell or Ward Counting Wheel, respectively. For quality assurance among taxonomists and between laboratories, a random 10% of all samples were re-identified by independent taxonomists. Any differences among taxonomists were reconciled before the final zooplankton dataset was compiled. Zooplankton abundance was estimated from the number of individuals counted and the volume of water sampled. Zooplankton biomass was estimated based on published, standard length-width relationships (references listed in Section 10.5, U.S. EPA 2012b).

We noted that some zooplankton species were counted in both the 50 and 150 *μ*m mesh samples. In this case, we averaged the results by sample location and taxon ID. For our analyses, we primarily focused on zooplankton biomass for the common members of the community. As with phytoplankton, we removed any zooplankton orders that constituted less than 5% of any sample. For the retained orders, we removed any genera that did not contribute at least 5% to at least one sample. These genus-level data were used for subsequent analysis, and aggregated to order-level as needed.

### Lake nutrient-color status assignment

We categorized lakes following the methods of Nürnberg and Shaw (1998) and Webster et al. (2008). Lakes were classified as oligotrophic or “blue” if TP concentration ≥ 30 *μ*g L^−1^ and true color ≤ 20 platinum cobalt units (PCU), eutrophic or “green” if TP > 30 *μ*g L^−1^ and true color ≤ 20 PCU, dystrophic or “brown” if TP ≤ 30 *μ*g L^−1^ and true color > 20 PCU, and mixotrophic or “murky” if TP > 30 *μ*g L^−1^ and true color > 20 PCU.

The detection limits for total phosphorus were 3.9 and 2.9 *μ*g L^−1^ in 2007 and 2012, respectively. All samples below this limit were flagged and removed from the NLA dataset by the EPA. Interestingly, no lakes were reported below detection limits in 2012 compared to 7% of samples in 2007 (Stoddard et al. 2016). True color was based on visual observations of water samples compared to a series of standards viewed through a color disk, with an estimated accuracy of 2.5 PCU (i.e., Hach Kit Model CO-1; EPA Method 110.2) (U.S. EPA 1987). Before analysis, water samples were passed through a 0.4 *μ*m Nucleopore filter and/or centrifuged to remove any turbidity before color determination. While this method involves a subjective analysis, all color measurements were made by the same lab technician in the same laboratory in both 2007 and 2012.

We did not use DOC concentration as an estimate of true color as it was noted that many lakes with high DOC concentrations in the NLA dataset had comparatively low true color values. These systems tended to have high Chl *a* concentrations, suggesting large inputs of non-chromophoric, algal-derived organic carbon. In addition, some lakes with high true color values had relatively lower DOC concentrations, suggesting that increases in true color were associated with inorganic inputs, such as iron or manganese. Spectral data (e.g., a_440_) are not collected as part of the NLA sampling program, and therefore, could not be used as a proxy for true color.

### Statistical analyses

All statistical analyses were performed in the R statistical environment (R Core Team 2017), with figures created using the “ggplot2” package (Wickham 2009). To assess potential shifts in lake nutrient-color status between 2007 and 2012, we first used the “spsurvey” package in R (Kincaid and Olsen 2016). We leveraged NLA’s stratified, randomized site selection process to develop statistically valid population estimates at continental and regional scales, using the entire datasets of lakes ≥ 4 ha in 2007 and 2012, equivalent to 1028 lakes in 2007 and 950 lakes in 2012. Population estimates were developed using a weighted Horvitz-Thompson estimation, with a local mean variance estimator to calculate 95% confidence intervals around the estimate for each assessment period (Lohr 1999; Stevens and Olsen 2003). We calculated change between the 2007 and 2012 populations at the national scale and within each of nine aggregate III ecoregions (Omernik 1987) using the change.analysis function in “spsurvey” (Kincaid and Olsen 2016).

We further analyzed shifts in lake nutrient-color status using the 401 lakes sampled in both 2007 and 2012, all of which were ≥ 4 ha in size. Unlike the dataset as a whole, these lakes were not purposefully selected to be representative of the entire population of lakes in the conterminous U.S. Nevertheless, we used them in our analysis to investigate changes in lake nutrient-color status within individual lakes over time. Each pair of points from each resampled lake was treated as a mathematical vector, calculating its magnitude and angle of direction. Using the “circular” package in R (Agostinelli and Lund 2017), we plotted the angles of direction on a rose diagram, calculated the mean angle of direction and bootstrapped the 95% confidence intervals with 999 permutations, and then performed a Rao Spacing Test to determine if the angles of direction were non-random. In addition, one-way ANOVAs were performed to assess potential differences in the angle of direction and magnitude of change between the four lake types. However, these ANOVA results should be assessed with caution given the differences in sample sizes: 204 blue lakes, 111 green lakes, 66 murky lakes, and 20 brown lakes (based on initial lake nutrient-color status in 2007).

We used the NLA 2012 dataset to investigate the effects of lake size and watershed characteristics on lake nutrient-color status as well as differences in food web structure across the four lake types. In total, there were 1013 lakes that fit our criteria for TP, color, phytoplankton, and zooplankton data. This set included 85 lakes 1–4 ha in surface area. Non-parametric tests were performed due to lack of normality and heteroscedasticity in the dataset. Given the overall number of statistical comparisons (i.e., 14 total Kruskal–Wallis tests described below), we adjusted alpha values using the Bonferroni correction.

Kruskal–Wallis tests in combination with the Dunn’s multiple comparison test, using the FSA package (Ogle 2017), were performed to determine whether lakes of varying nutrient-color status differed in the percentage of urban development, agriculture, forest cover, and wetlands within their watersheds as well as in total watershed area and lake area. To assess basal pelagic production, we used Kruskal–Wallis–Dunn tests to detect significant differences in Chl *a* concentrations, total phytoplankton biomass, the density of blue-green algae, and the concentration of microcystin toxin. To assess zooplankton production, we also used Kruskal–Wallis–Dunn tests to detect potential significant differences in zooplankton biomass with lake type, including comparisons of rotifer, copepod, cladoceran, and total biomass.

To visualize potential differences in zooplankton community structure associated with differences in lake nutrient-color status, we used non-metric multidimensional scaling (NMDS), both at the order- and genus-level. Permutational multivariate analysis of variance (PERMANOVA) was then used to test for significant differences in zooplankton community composition across lake nutrient-color status. Both the NMDS and PERMANOVA were run based on zooplankton density and biomass, using the “vegan” package in R (i.e., metaMDS and adonis functions, respectively) (Oksanen et al. 2017). The metaMDS automatically applies a square-root transformation and calculates Bray–Curtis dissimilarity distances.

Because many data points occurred near the intersection of the lake type benchmarks, we further investigated differences in zooplankton community biomass in “extreme” lakes within each nutrient-color lake type. Extreme was defined as follows: for blue lakes, values below first quartile for both TP (10 *μ*g L^−1^) and color (7.5 PCU); for green lakes, below first quartile for color (11 PCU) and above third quartile for TP (124 *μ*g L^−1^); for brown lakes, values above third quartile for color (44 PCU), and below first quartile for TP (15 *μ*g L^−1^); and for murky lakes, values above third quartile for both TP (302 *μ*g L^−1^) and color (38 PCU).

## Results

### Shifts in lake nutrient-color status: population estimates

**Fig. 1.**
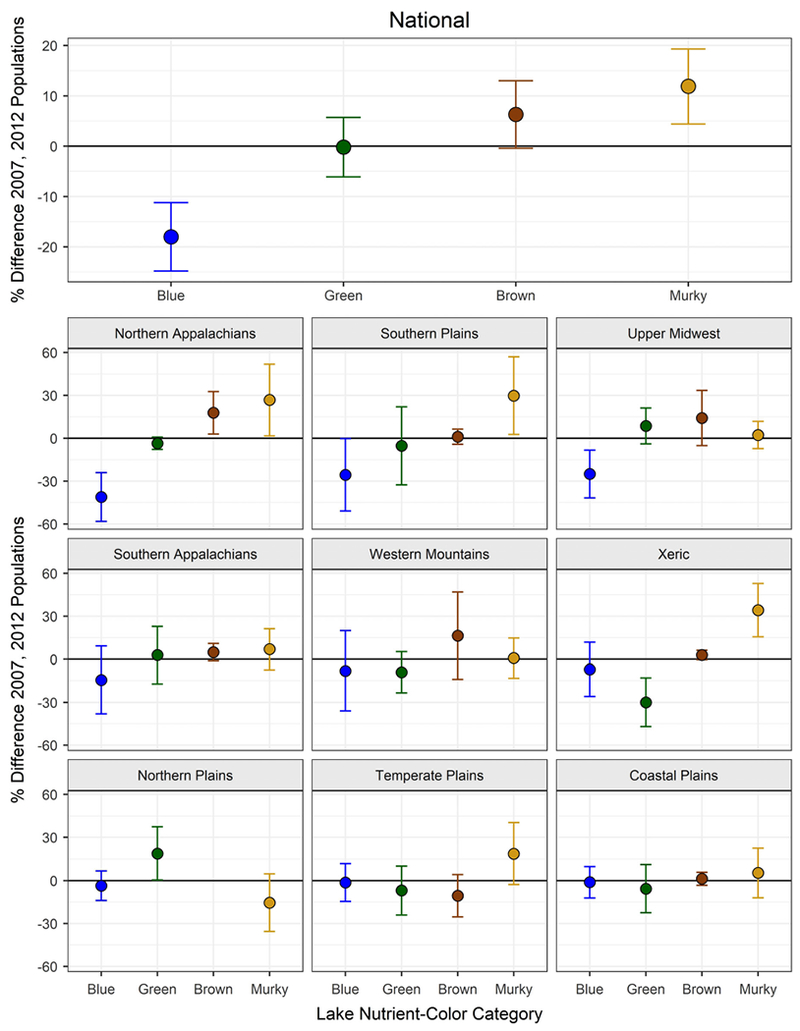
Change in nutrient-color status across the population of lakes ≥4 ha in the continental U.S. (national) and in nine aggregated Omernik level-III ecoregions (*n* = 1028 in 2007 and *n* = 950 in 2012). Each point represents the percent difference in the population within a nutrient-color category between 2007 and 2012. The population of lakes within a category can increase (above zero), remain the same, or decrease (below zero). The bars around the point represent the 95% confidence interval associated with that change. A change is statistically significant if the 95% confidence interval does not include zero.

At the continental-scale of the U.S., the proportion of blue lakes significantly decreased by 18%, from 45.7% of the population of lakes in 2007 to 27.7% of the population of lakes in 2012 (Table 1; Fig. 1). In contrast, the proportion of murky lakes increased by ~ 12%, from 23.5% in 2007 to 35.4% of the population in 2012 (Table 1; Fig. 1). There were no statistical differences in the proportions of brown or green lakes from 2007 to 2012 (Table 1; Fig. 1). Geographically, when the lakes were analyzed within each of the nine Omernik III ecological regions, we observed that: (1) in the Northern Appalachians, blue lakes decreased by 41.4%, brown lakes increased by 17.8%, and murky lakes increased by 26.8%, (2) in the Northern Plains, green lakes significantly increased by 18.9%, (3) in the Southern Plains, blue lakes significantly decreased by 25.6% and murky lakes increased by 29.8%, (4) in the Upper Midwest, blue lakes significantly decreased by 25%, and (5) in the Xeric region, green lakes significantly decreased by 30% while murky lakes increased by 34.2% (Table 1; Fig. 1). No significant changes were observed in the Coastal Plains, Southern Appalachians, Temperate Plains, or Western Mountains ecoregions (Table 1; Fig. 1). By 2012, murky lakes represented the most abundant lake type in the dataset (~ 35%), followed by blue lakes (~ 28%), green lakes (~ 27%), and brown lakes (~ 10%), based on 1013 lakes (i.e., includes lakes 1–4 ha).

### Shifts in lake nutrient-color status: temporal trends in resampled lakes

**Fig. 2.**
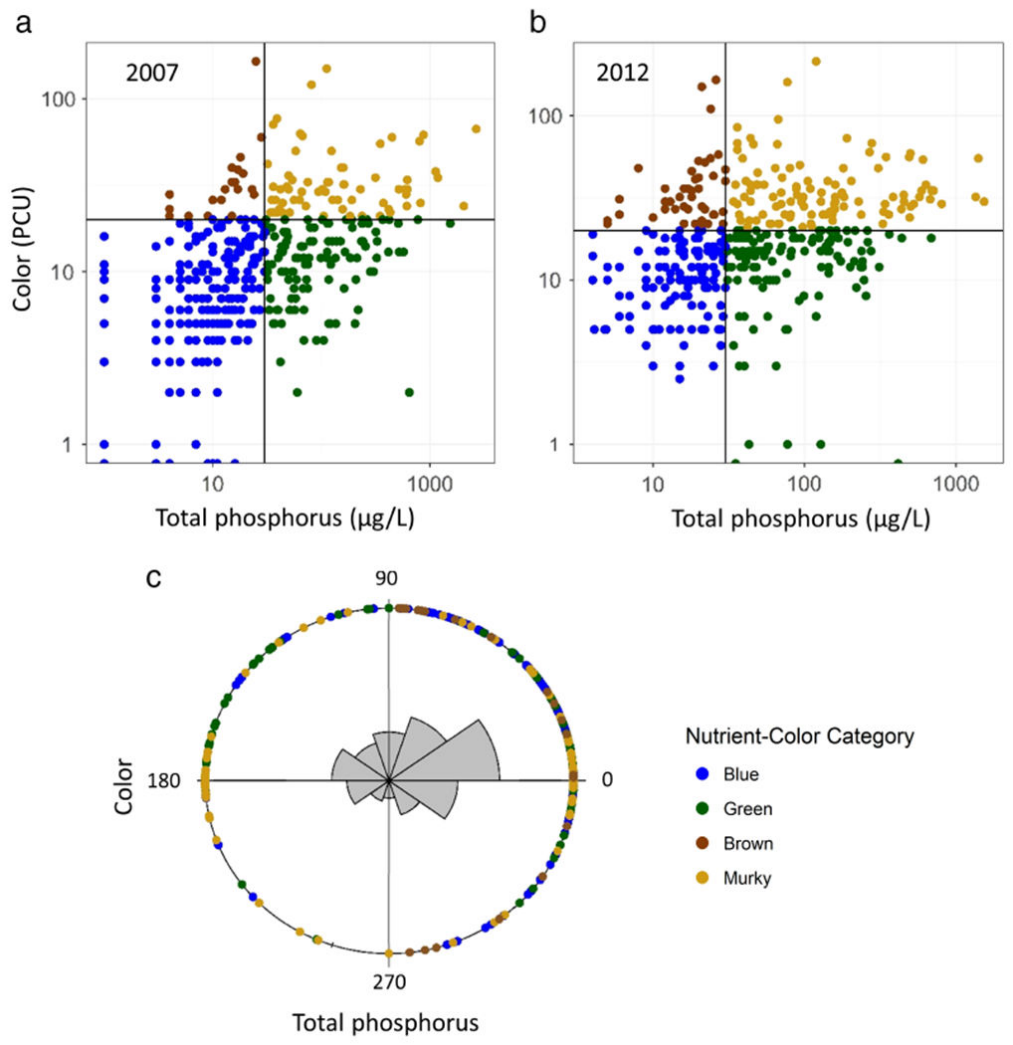
(**a-c**) Nutrient-color status of lakes in 2007 (**a**) and 2012 (**b**) and the direction of change between the two sampling years (**c**), based on the 401 resampled lakes in the NLA datasets. Panel C is a rose diagram of the angle of direction for the vector between each pair of points (i.e., a lake’s TP and color concentration in 2007 and 2012). Total phosphorus is on the *x*-axis and water color on the y-axis. The mean angle of direction was 32.68°, with a 95% confidence interval of 26.36°−39.53°, suggesting that most lakes increased in TP and color between the two NLA surveys. The colored dots around the circle depict the angle of direction for each individual lake. Colors represent a lake’s nutrient-color status in 2007. Note that fewer dots are located in the lower right quadrant, or toward blue (i.e., decreasing in TP and color).

**Fig. 3.**
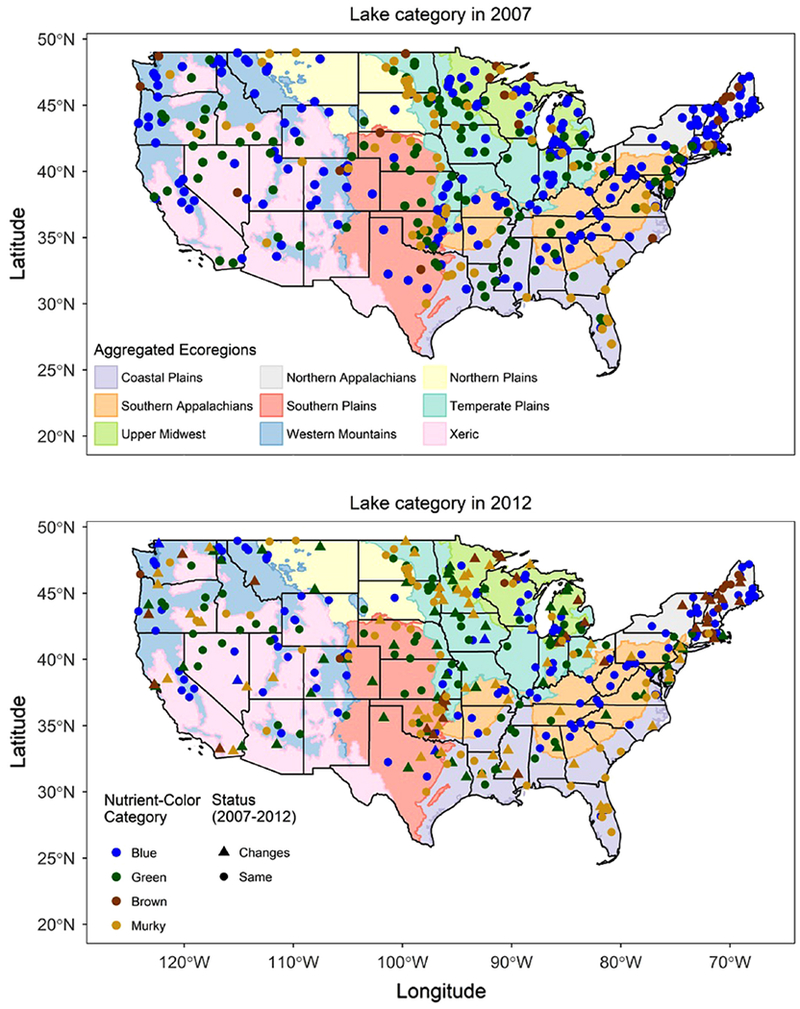
Location and nutrient-color status of the 401 lakes sampled in both the 2007 and 2012 NLA surveys overlaid on the nine Omerick III ecoregions across the U.S. nutrient-color status for all 401 lakes in 2007 are shown in the top panel; the bottom panel distinguishes lakes which changed nutrient-color status from those that remained the same.

Examining only the 401 resampled lakes, 64.8% remained in the same lake class and 35.2% changed class between 2007 and 2012 across the U.S. (Figs. 2a,b, 3). For the lakes that changed class, most shifted to green or murky status (Table 2). The mean angle of direction of change for all resampled lakes was 32.68° with a 95% confidence interval of 26.36°ߝ39.53° (Fig. 2c). For reference, an angle of 45° would indicate that a lake was increasing in equal proportions of TP and color. Thus, both total phosphorus and color increased in the majority of resampled lakes between 2007 and 2012, with proportionally greater increases in TP. The Rao spacing test confirmed that these shifts were significantly different from random (T-statistic = 204.47, *p* < 0.001). There were no significant differences in the angle of direction among the four lake classes (*F* = 2.013, df = 3, *p* = 0.11). However, there were significant differences in magnitude between lake classes (*F* =17.11, df = 3, *p* < 0.001), with murky lakes exhibiting the largest magnitudes of change (median = 59.54; range = 2–2364) and blue lakes exhibiting the smallest (median = 15.18; range = 1.3–166). The median magnitude of change for green lakes was 37.33 (range = 2.8–534) and 22.66 for brown lakes (range = 2.2–126). The large magnitude of change in murky lakes rarely led to a shift in nutrient-color status. Only 10 murky lakes displayed shifts: 8 to green, 2 to brown, and 0 to blue (Table 2).

**Fig. 4.**
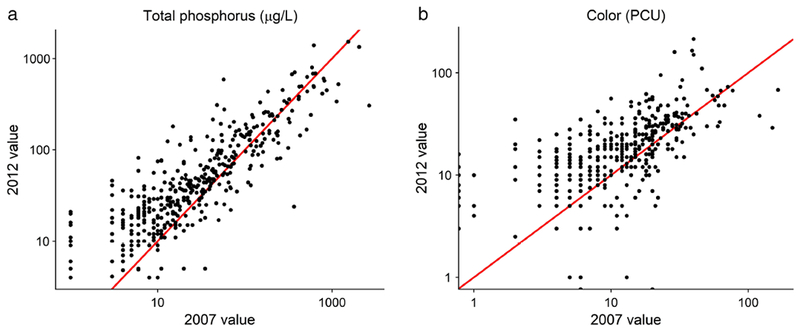
Comparison of total phosphorus concentrations (**a**) and true color (**b**) in the 401 lakes sampled during both the 2007 and 2012 NLA surveys plotted on a log scale. The red line represents the 1 : 1 ratio or zero change. Most lakes were observed to increase in total phosphorus and color between 2007 and 2012.

Overall, 75% of the resampled lakes increased in TP concentrations, 73% increased in color, and 56% increased in both TP and color between 2007 and 2012 (Fig. 4). Changes in total phosphorus in blue lakes ranged from −15.0 *μ*g L^−1^ to +166 *μ*g L^−1^ between 2007 and 2012 whereas changes in color ranged from −13.0 PCU to +44.0 PCU (Table 2). These increases often tipped lakes from blue to green, brown, or murky (Table 2; Fig. 2a–c) and account for the decrease in blue lakes since 2007. In green lakes, changes in total phosphorus ranged from −360 *μ*g L^−1^ to +534 *μ*g L^−1^ whereas changes in color ranged from −20.0 PCU to +46.0 PCU between 2007 and 2012. Consequently, many lakes classified as green in 2007 became murky by 2012 (Table 2; Fig. 2c). Within brown lakes, total phosphorus only increased, ranging from +2.0 *μ*g L^−1^ to +104.0 *μ*g L^−1^, while changes in color ranged from −97.0 PCU to +126.0 PCU. Murky lakes had the widest range of change in total phosphorus, from −2364 *μ*g L^−1^ to +780 *μ*g L^−1^ (Table 2). Given the initially high TP concentrations for these lakes, the impact on lake classification was negligible. Changes in color in murky lakes ranged from −121 PCU to +174 PCU (Table 2).

### Land use/land cover, watershed size, and lake class

When examining percent land cover in the 2012 NLA dataset, blue and brown lakes had significantly more forest within their watersheds (KW chi-squared (*χ*^2^) = 157.87, df = 3, *p* < 0.0001) while green and murky lakes had a greater percentage of agriculture (KW *χ*^2^ = 85.663, df = 3, *p* < 0.0001) (Table 3). Brown and murky lakes had significantly more wetlands within their watersheds compared to blue and green lakes (KW *χ*^2^ = 55.527, df = 3, *p* < 0.0001) (Table 3). No significant differences in urban development were observed across the lake types (Table 3). In terms of total watershed area, blue and green lakes possessed significantly larger watersheds compared to brown and murky lakes in the 2012 NLA dataset (KW *χ*^2^ = 23.51, df = 3, *p* < 0.0001) (Table 3).

### Lake area and lake class

Lake area significantly differed with nutrient-color status (KW *χ*^2^ = 23.69, df = 3, *p* < 0.0001), with blue and green lakes significantly larger in surface area than brown and murky lakes. The median size of blue and green lakes was ~ 40 ha while that of brown and murky lakes was ~ 22 ha (Table 3). However, these results should be interpreted with caution. Frequency distributions of lake size revealed that most lakes were between 1 to 10 ha in surface area for all four lake classes. (In the 2007 NLA survey, most lakes were between 4 and 10 ha in size in each lake class.) Of the 85 lakes between 1 and 4 ha included in the 2012 NLA dataset, 21 were classified as blue (25%), 21 green (25%), 8 brown (9%), and 35 murky (41%). Thus, all four nutrient-color classes included lakes smaller in size, and the largest lake included the 2012 NLA dataset (~ 167,000 ha) was classified as murky (Table 2). Brown lakes displayed the narrowest range in lake area (i.e., 1–4213 ha, Table 3).

### Basal pelagic resources

**Fig. 5.**
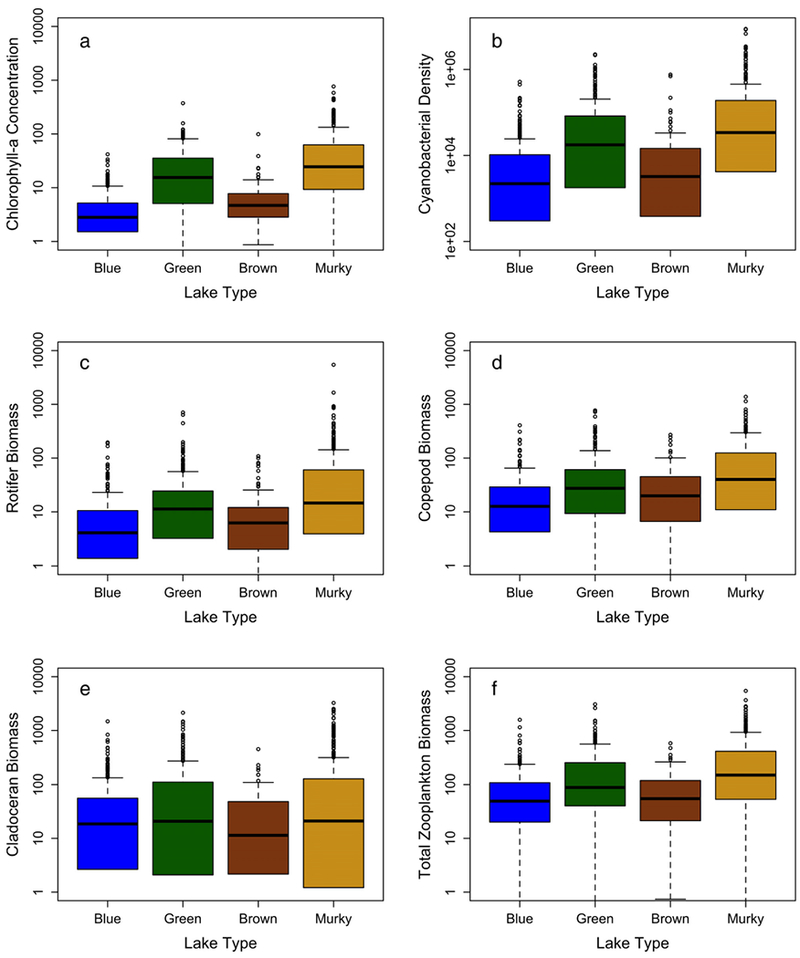
Boxplots of Chl *a* concentration (μg L^−1^) (**a**), cyanobacterial density (cells mL^−1^) (**b**), rotifer (**c**), copepod (**d**), cladoceran (**e**), and total zooplankton (f) biomass across the four lake types. Zooplankton biomass is in units of μg dry weight L^−1^. All plots are displayed on a log-scale with boxes color-coded by lake class, from blue to murky. The line in each box represents the median, the box represents the first and third quartiles, and the whiskers represent the lower and upper extremes. Outliers are shown as circles. Significance is listed in Table 4.

Based on the 2012 NLA survey, Chl *a* concentrations significantly differed among the lake types (KW *χ*^2^ = 361.21, df = 3, *p* < 0.0001), with higher concentrations in murky lakes (median = 24.48 *μ*g L^−1^, range = 0.22–764.6 *μ*g L^−1^) followed by green lakes (median = 13.27 *μ*g L^−1^, range = 0.33–373.3 *μ*g L^−1^) (Tables 3–4; Fig. 5a). Moreover, the density of cyanobacteria significantly differed among lake types (KW *χ*^2^ = 200, df = 3, *p* < 0.0001) and was highest in murky lakes (median = 34,210 cells mL^−1^, range = 0–8,757,000 cells mL^−1^) followed by green lakes (median = 14,460 cells mL^−1^, range = 0–2,245,000 cells mL^−1^) (Tables 3–4; Fig. 5b). The concentration of microcystin toxin was also significantly higher in murky lakes (median = 0.13 *μ*g L^−1^, range = 0–66.7 *μ*g L^−1^) followed by green lakes (median = 0.10 *μ*g L^−1^, range = 0–42.2 *μ*g L^−1^; KW*χ*^2^ = 100.16, df = 3, *p* < 0.0001) (Table 3). Blue and brown lakes did not significantly differ in Chl *a* concentrations, cyanobacterial density, or microcystin concentration (Tables 3–4; Fig. 5a,b). Despite increased phytoplankton and zooplankton biomass in murky lakes, the ratio of zooplankton to phytoplankton biomass was similar to that in green lakes and significantly lower than that in blue and brown lakes (KW *χ*^2^ =27.159, df = 3, *p* < 0.0001) (Table 3).

### Zooplankton biomass and community structure

**Fig. 6.**
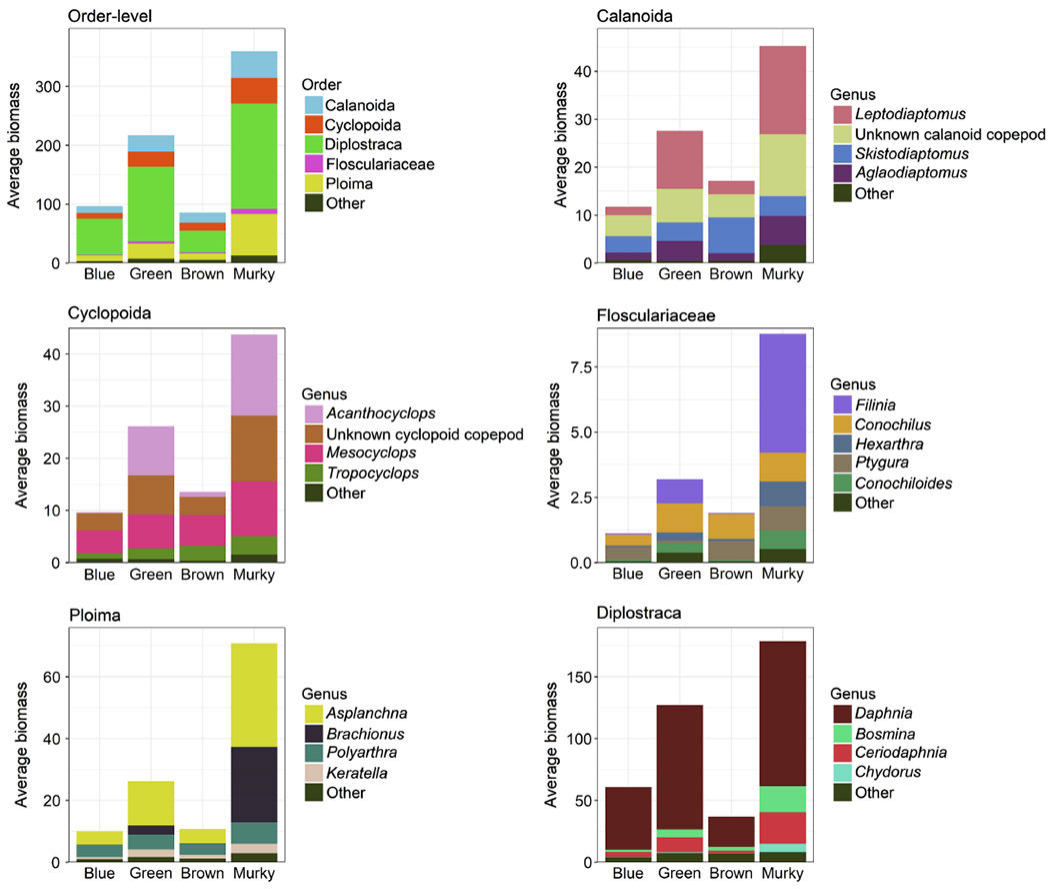
Average zooplankton biomass by order-level and genera-level across lake nutrient-color status, based on data from the 2012 NLA. Orders and genera contributing less than 5% of any sample were removed; order-level biomass is the sum total of all remaining genera within an order. Plot includes 1013 lakes total: 284 blue, 273 green, 109 brown, and 347 murky. Note differences in *y*-axis limits.

Total zooplankton biomass significantly differed with lake type in the 2012 NLA dataset (KW *χ*^2^ = 104.91, df = 3, *p* < 0.0001), with the highest biomass observed in murky lakes (median = 150.04 *μ*g dry weight L^−1^, range = 0.16–5448.9 *μ*g dry weight L^−1^) followed by green lakes (median = 88.37 *μ*g dry weight L^−1^, range = 0.18–3069.62 *μ*g dry weight L^−1^) and no difference in blue (median = 49.18 *μ*g dry weight L^−1^, range = 0.13–1579.85 *μ*g dry weight L^−1^) and brown lakes (median = 54.66 *μ*g dry weight L^−1^, range = 0.74–578.73 *μ*g dry weight L^−1^) (Table 4; Fig. 5f). Diplostraca, especially *Daphnia* spp., were the predominant contributors to zooplankton biomass in all four lake types (Fig. 6a,f; Supporting Information Table S1), and there were no differences in Diplostraca biomass across lake types (Fig. 5e; Table 4). Calanoids and cyclopoids constituted relatively equal proportions of biomass within each lake class (e.g., ~ 13% and 12%, respectively, in green lakes; ~ 13% and 12%, respectively, within murky lakes) (Fig. 6a-c;Supporting Information Table S1). However, copepod biomass significantly differed across lake types, with the highest copepod biomass observed in murky lakes, followed by green, blue, and brown lakes (Fig. 5d; Table 4).

Although they represent a smaller proportion of the total biomass, the most notable differences in zooplankton among the lake types were observed within the rotifers (Fig. 6a-f). Rotifer biomass was significantly greater in murky lakes compared to the other lake types (Fig. 5c). The average compositional biomass of Ploima in murky lakes was nearly double that in blue lakes (i.e., ~ 20% vs. 10%, respectively) (Supporting Information Table S1). One-third of the Ploima biomass in murky lakes was comprised of *Brachionus* spp. compared to ~ 11% in green lakes and ~ 1% in blue and brown lakes (Fig. 6e). *Asplanchna* spp. also displayed higher biomass in murky lakes compared to the other lake types (Fig. 6e). Flosculariaceae contribute the least average biomass among the major zooplankton orders across all lake types but was again relatively higher in the murky lakes (Fig. 6a; Supporting Information Table S1). This related overwhelmingly to an increase in *Filinia* and *Hexarthra* biomass in murky lakes (Fig. 6d).

Despite these differences in rotifer and copepod biomass, multivariate approaches did not reveal significant differences in zooplankton community composition at the order-, genus-, or species-level related to lake nutrient-color status when all data points were included in the analyses (NMDS stress values ≥ 0.2; PERMANOVA *R*^2^ ≤ 0.05). When only the most extreme lakes were examined within each of the four lake types—for example, those murky lakes with TP and color values both in the upper quartiles and blue lakes with values in the lower quartiles—average zooplankton biomass more than doubled in murky lakes (Supporting Information Fig. S1), which are located primarily in the central U.S. (Supporting Information Fig. S2). Based on PERMANOVA, zooplankton communities in extreme murky lakes differed significantly from communities in extreme blue lakes (Pseudo-*F* = 10.91, df = 1, *p* = 0.001), with lake nutrient-color status accounting for nearly 18% of the observed variance (*r*^2^ = 0.176). Diplostraca continued to dominate the zooplankton biomass of murky lakes, with *Daphnia* spp. comprising the majority (60%) of Diplostraca present. In addition, an increase in *Acanthocyclops* proportion contributed to the higher cyclopoid biomass in extreme murky lakes. Zooplankton composition was nearly identical across all lakes when extreme lakes are excluded from each lake type (Supporting Information Fig. S3).

## Discussion

Based on data from the EPA National Lakes Assessment, we demonstrate that an increasing proportion of lakes in the continental U.S. are simultaneously experiencing eutrophication (aka “greening”) and brownification (aka “browning”), such that murky lakes dominated the 2012 NLA survey. Once murky, our analysis further shows that it has not been common for lakes to shift back to simply brown or green. From a food web perspective, this shift in lakes toward murkiness promotes increased phytoplankton and zooplankton biomass. However, the low ratio of zooplankton to phytoplankton biomass suggests a reduction in energy transfer to higher trophic levels.

### Shifting lake nutrient-color status

Between 2007 and 2012, there was a significant reduction in blue lakes and a significant increase in murky lakes in the continental U.S., particularly in the Northern Appalachian and Southern Plains ecoregions, based on population estimates from the NLA surveys. Moreover, analyzing the direction of change in the 401 resampled lakes revealed increases in both TP concentrations and color in over half of this subset of lakes. While our findings were similar from these two approaches, there were slight differences in the reported proportions of lakes changing nutrient-color status. This is likely due to two major factors. First, there were differences in sample sizes. The population analysis was developed from the full set of lakes ≥ 4 ha sampled in 2007 and 2012 (*n* = 1028 and 950, respectively) while the resampled lake analysis included only 401 lakes. Second, the approaches represent different aspects of change. The national analysis represents changes across the population of lakes ≥ 4 ha in the continental U.S. Due to NLA’s statistically representative site selection process, each sampled lake is weighted by its frequency of occurrence on the landscape, and this is used to develop statistically valid inferences from the sampled lakes to a target population of lakes in the continental U.S. The national change analysis compared the inferred 2007 and 2012 population-level results. In contrast, the 401 resampled lakes were not a representative subsample of the probability design. Rather, these lakes allowed us to examine changes in nutrient-color status within individual lakes and to start an initial exploration of the range of TP and color change experienced by lakes over time. While slightly different, these two analytical approaches demonstrated shifts in nutrient-color status both at the national scale of the population-level inferences, and within individual lakes.

Overall, these results suggest the continued presence of both lake greening and browning on a national scale. We cannot definitively say what is causing lakes to shift in nutrientcolor status (i.e., increase in TP and/or color), but land cover and land use patterns within a watershed often play a role. Current lake water quality reflects longer-term influences of a watershed. For example, the prevalence of agriculture within a lake basin is often linked to lake greening (Moss 2008; Schindler et al. 2016). This possibility is suggested in the NLA data, where green and murky lakes have a significantly higher percentage of agricultural land cover within their watersheds compared to blue and brown lakes. Increased runoff of nitrogen and phosphorus fertilizer from agricultural fields can dramatically stimulate phytoplankton growth, especially blooms of filamentous blue-green algae that can cover the surface of lakes (Heisler et al. 2008; Smith and Schindler 2009). Lake browning, on the other hand, is often associated with the prevalence of wetlands within a watershed (Larsen et al. 2011). Again, NLA data support these prior observations, with brown and murky lakes having a greater percentage of surrounding wetlands compared with blue and green lakes. Decomposition of organic matter is substantially reduced in wetlands due to a lack of dissolved oxygen. Consequently, wetlands often contribute increased inputs of CDOM to nearby lakes that stain water dark brown.

These observed patterns in land cover and use also coincide with regional patterns in lake nutrient-color status. The proportion of blue lakes significantly declined in ecoregions of the central U.S. but remained prominent at higher elevations in the Eastern and Western mountains. In contrast, green and murky lakes were prominent in the central and southeastern U.S. but occurred less frequently in mountainous regions. The central and southeastern U.S. are not only characterized by extensive agriculture but also include many types of wetland habitats (U.S. Fish and Wildlife Service 2011). Both have the potential to contribute to nutrient and organic matter loading (Dalzell et al. 2005; Solomon et al. 2015), leading to greener or murkier lakes. The brownification of blue lakes was especially evident in the northeastern U.S., which also has an abundance of wetland habitats—for example, 25% of the land area in Maine is categorized as wetland (US Fish and Wildlife Service 2011).

Changes in climate are also likely to influence shifts in lake nutrient-color status. A detailed, regional assessment of temperature and precipitation patterns in 2007 vs. 2012 for lakes in the NLA was beyond the scope of this study, but the importance of climate change must be acknowledged. Increased temperatures have been shown to increase soil DOM concentrations, and depending on hydrologic conditions, can lead to increased organic matter runoff to nearby inland waters (Freeman et al. 2001; Nguyen and Choi 2015). Moreover, increased precipitation, especially extreme events, is often linked to the brownification and eutrophication of lakes (Williamson et al. 2015; de Wit et al. 2016; Carpenter et al. 2018). In the present study, for example, the brownification of blue lakes in the northeastern U.S. may be facilitated by longer-term regional changes in climate; a 27% increase in precipitation and > 1.5°C increase in annual temperature has been observed here since 1901 (U.S. GCRP 2017). Nevertheless, the browning of lakes in this region may also be due to reductions in acid precipitation, which was occurring within the same timeframe (Monteith et al. 2007). Further analyses of shifting lake nutrient-color status with local and regional climate, as well as other atmospheric changes, are needed. Lakes in the NLA survey are sampled only once during the summer, but it is possible that lakes shift in nutrient-color status multiple times throughout a given year, or season.

Finally, brown and murky lakes were observed to be smaller in surface area than blue and green lakes. Lakes smaller in surface area tend to be shallower in depth, and therefore, have a smaller volume (Cael et al. 2017). Consequently, smaller lakes likely require less allochthonous inputs to cause major changes in lake TP and organic matter concentrations. Nevertheless, while statistically significant, we are not completely convinced that lake area is an important variable in determining lake nutrient-color status. Most lakes in all four of the lake classes were 1–10 ha in size, which is reflective of the global size distribution of lakes (Downing et al. 2006; Cael and Seekell 2016). Not surprisingly, many of the larger lakes in the 2012 NLA survey were classified as blue. However, the fact that there also were several large green lakes in the 2012 NLA survey highlights the effects of 100+ years of eutrophication across the continental U.S. The complex relationship between size, geographic local, climate, and lake nutrient-color status warrants further investigation.

### Lake nutrient-color status and water quality

From a societal perspective, shifts in lake nutrient-color status can alter lakes in a manner that leads to nuisance or hazardous conditions. Some cyanobacteria, like *Microcystis* and *Anabaena* spp., produce toxins that can cause damage to the liver and nervous system as well as liver and colon cancer (Zanchett and Oliveira-Filho 2013; Wood 2016). In the present study, the highest recorded concentrations of the toxin microcystin occurred in murky lakes. Encouragingly, only 14 lakes in the 2012 NLA survey exceeded the 10 *μ*g L^−1^ guidelines for recreation waters: 7 murky, 6 green, and 1 blue lake. However, ~ 14% of murky lakes and ~ 12% of green lakes exceeded the 1 *μ*g L^−1^ provisional guideline set by the World Health Organization for finished drinking water. In contrast, < 1% of blue and 3% of brown lakes exceeded this limit. Both increased microcystin and cDOM concentrations in drinking water reservoirs pose technical challenges during drinking water purification (Hoeger et al. 2005; Chow et al. 2007).

Controlling the quantity and quality of allochthonous organic matter inputs from the watershed may be near impossible, particularly with respect to continued changes in global temperatures and precipitation patterns (Freeman et al. 2001; de Wit et al. 2016). However, attention to agricultural activity in the watershed may help stabilize or reverse changes in lake nutrient-color status, and thereby improve water quality. Yet a recent study by McCrackin et al. (2017) suggests that lakes are slow to, or may not, recover from eutrophication even after complete cessation of external nutrient loading. This is concordant with previous studies that show high variability in results of “reoligotrophication” efforts (Jeppesen et al. 2005). Resistance to recovery may be related to ongoing internal loading of phosphorus from lake sediments caused by anoxic conditions in the hypolimnion. The latter is often exacerbated by lake greening and browning (Solomon et al. 2015; Williamson et al. 2015; McCrackin et al. 2017).

### Pelagic basal resources across lake types

Phytoplankton biomass increased across the lake types from blue to murky, coinciding with increases in TP concentration and/or water color. Increased phosphorus inputs have long been known to stimulate primary production. However, recent studies have demonstrated that organic matter inputs can also fuel increased algal growth, particularly in nutrientlimited systems with low DOC concentrations (Klug 2002; Cooke et al. 2015; Seekell et al. 2015; Williamson et al. 2015; Fergus et al. 2016). This increase in primary production is believed to be associated with nutrient additions and UV protection acquired from increasing organic matter inputs.

As lakes continue to darken in brown color, primary production has been shown to decrease due to the reduction in light for photosynthesis (Ask et al. 2012; Thrane et al. 2014; Solomon et al. 2015). Here, surprisingly, the increased color of murky lakes did not appear to have an inhibitory effect on epilimnetic phytoplankton, as they displayed the highest phytoplankton biomass. The shallowing of the mixed layer with increased color may enable photosynthetic cells access to light in the surface waters despite an overall reduction in light penetration through the water column (Solomon et al. 2015). Moreover, a relatively large proportion of algal biomass in murky lakes was composed of cyanobacteria, which often create mats across the surface of lakes (Heisler et al. 2008; Smith and Schindler 2009). Increased nutrient loading associated with organic matter inputs, in combination with inorganic phosphorus loads, may further fuel primary production in murky lakes compared to the other lake types.

In terms of bioavailability, however, the proportion of inedible phytoplankton has been shown to increase with lake trophy (Watson and Kalff 1981; Jeppesen et al. 2000; Hessen et al. 2003; Heathcote et al. 2016). Here, densities of blue-green algae and concentrations of microcystin toxin were high in green, and especially, murky lakes, which may deter zooplankton feeding (Gliwicz and Lampert 1990; Leflaive and Ten-Hage 2007). This inference is supported by the low zooplankton : phytoplankton biomass ratio observed in green and murky lakes. Low zooplankton to phytoplankton biomass ratios suggest a reduction in energy transfer efficiency through the food web. According to Downing et al. (2001), cyanobacteria account for an average of 60% of phytoplankton biomass at TP concentrations above 80–90 *μ*g L^−1^, and Jeppesen et al. (2000) found that herbivorous zooplankton consume 50% of algal biomass per day in lakes of low trophy (<50 *μ*g PL^−1^) but only 16–19% at higher trophy (200–400 *μ*g P L^−1^). Compared to other algal taxa, cyanobacteria have lower concentrations of key fatty acids, such as DHA and EPA (Taipale et al. 2016). Thus, while phytoplankton biomass was high in green and murky lakes, a relatively large portion may represent a non-viable or less nutritious resource to higher trophic levels.

### Zooplankton community structure across lake types

As predicted, blue and brown lakes had significantly lower total zooplankton biomass compared to green and murky lakes, which coincided with differences in phytoplankton biomass across the lake types. Murky lakes displayed the highest zooplankton biomass, primarily due to an increase in rotifer and copepod biomass. Typically, eutrophic systems are cited as having high rotifer biomass (Pace 1986); however, here we show that mixotrophic “murky” systems support even greater rotifer biomass. Predatory copepods, both calanoid and cyclopoid, may in turn benefit from the increase in rotifer biomass (Brandl 2005).

The rotifers *Asplanchna, Filinia, Hexartha,* and *Brachionus* spp. were particularly abundant in murky lakes. While *Asplanchna* can be omnivorous and predatory, these other rotifers are known to be at least partially and potentially entirely bacterivorous (Starkweather et al. 1979; Sanders et al. 1989; Ooms-Wilms et al. 1995). Some macrozooplankton can also consume bacteria, and in some cases, a combined algalbacteria diet enhances growth rates (e.g., *Daphnia,* Freese and Martin-Creuzburg 2013). Although we do not have estimates of bacterial abundance or biomass for the NLA lakes, bacterial densities generally increase with increasing food resources, including increased primary production and organic matter concentrations (Cole et al. 1988; Wetzel 2001). Zooplankton in murky lakes may therefore have the option to feed on an abundance of both algae and/or bacteria (Jones 1992; Moore et al. 2004).

Despite high zooplankton biomass, zooplankton to phytoplankton biomass ratios were lower in green and murky lakes compared to blue and brown lakes. We further investigated crustacean (i.e., copepods and cladocerans) vs. rotifer biomass to determine whether increased rotifers in green and murky lakes could explain the lower zooplankton : phytoplankton biomass ratios. However, median rotifer to phytoplankton biomass ratios were similar in all lake types (~ 0.02–0.03) while median crustacean zooplankton to phytoplankton biomass ratios were higher in blue (~ 0.24) and brown (~ 0.17) lakes compared to green and murky lakes (~ 0.08). These results provide further supporting evidence that the low zooplankton : phytoplankton biomass ratios in green and murky lakes are related to increases in inedible algae, reducing energy transfer to higher trophic levels.

### Fish and lake nutrient-color status

It is possible that low zooplankton to phytoplankton biomass ratios in green and murky lakes are due to increased fish predation (Jeppesen et al. 2000; Hessen et al. 2003). Unfortunately, the NLA dataset does not provide information on fish communities. In general, fish biomass increases with lake productivity, with a shift to more planktivores compared with piscivores (Jeppesen et al. 2000). However, the combination of organic matter and nutrient inputs may reduce fish biomass. Murky lakes, in particular, may provide less usable habitat for fish given lower light and dissolved oxygen levels, particularly at deeper depths (Stasko et al. 2012). Karlsson et al. (2015) noted a decrease in fish biomass with increasing lake DOC concentration, which they equated with an increase in lake color. Similarly, Craig et al. (2017) recently reported that bluegill from humic lakes were smaller in body size and had reduced fecundity compared to those in clear lakes. Taipale et al. (2016) noted that perch from lakes with high phosphorus and dissolved organic carbon concentrations generally had smaller body sizes, which correlated with lower concentrations of nutritional EPA and DHA fatty acids. Thus, despite the increase in basal resources in murky lakes, the quality of food for higher trophic levels may be substantially less, leading to slower growth rates and poorer nutritional quality in fish. From a lake management perspective, this presents a challenge for using trophic cascades to biologically control algal blooms (Carpenter et al. 2001).

### Potential caveats

We acknowledge that these results are based on one time point for each lake collected during the summer. As mentioned above, it is possible that lakes exhibit seasonal shifts in lake nutrient-color status as nutrient and organic matter inputs fluctuate throughout the year. Interannual fluctuations in weather (e.g., drought) are also likely influential, and caution must be applied here when interpreting long-term trends based on two time points collected 5 yr apart. Further exploration of seasonal and interannual patterns in lake nutrientcolor status is indeed warranted, and suitable data may be available in long-term lake monitoring programs. We encourage future research in this area as management strategies to improve or maintain ecosystem health will likely differ depending on lake nutrient-color status.

In addition, zooplankton samples were only collected during the day and may therefore underestimate the density and biomass of large cladocerans and copepods exhibiting diel vertical migration. This is particularly true for lakes deeper than 5 m where essentially only the surface waters were sampled (US EPA 2012b). Large zooplankton are especially likely to avoid the surface waters of blue lakes during daylight in avoidance of damaging UV radiation (Leech and Williamson 2001) and visually feeding predators. Seasonal differences in zooplankton community structure may also have influenced our results given that the NLA sampling occurred from June through September. Future studies related to the consequences of lake nutrient-color changes need to consider the full spectrum of zooplankton as well as effects on seasonal shifts in community composition and structure.

## Conclusions

Murky lakes are increasing nationwide in the continental U.S., which presents many challenges for lake management. Our results indicate that water quality and lake food webs within each of the four lake types function differently. If these differences are not taken into account, it will be difficult to make realistic predictions as to how lakes will respond to future perturbations, such as climate warming and other anthropogenic impacts. Rather than focusing separately on eutrophication or brownification, more research is needed to understand how the combined “greening” and “browning” of lakes affects ecological processes within murky systems and to develop potential strategies that reverse their nutrient-color status.

## Supplementary Material

Figure S1

Figure S2

Figure S3

Table S1

## Figures and Tables

**Table 1.** Number of lakes classified in each lake nutrient-color category during the 2007 and 2012 NLA surveys (*n* = 1028 and 950 lakes, respectively), and in parentheses, the estimated percent of lakes on the landscape represented by the surveyed lakes (± standard error [SE]) within an ecoregion and at the national scale. These combined values were used in the population estimate analysis to determine shifts in lake nutrient-color status at the national and regional scale between 2007 and 2012. Ecoregions represent the nine aggregate III ecoregions designated by the EPA. No lakes were classified as “brown” in the Northern Plains in either 2007 or 2012. Only lakes > 4 ha were included in the analysis, which reduced the total number of lakes in the 2012 dataset.

	Blue		Green		Brown		Murky	
	2007	2012	2007	2012	2007	2012	2007	2012
*Ecoregion*								
Coastal plain	20 (19.8±4.5)	17 (18.7±5.4)	37 (29.4±6.7)	30 (23.9±5.8)	5 (3.9±3.9)	10 (5.3±5.3)	39 (46.8±7.2)	59 (52.1 ±6.9)
Northern Appalachian	64 (75.1 ±4.9)	36 (34.0±7.7)	11 (8.7±2.3)	6 (5.2±2.1)	15 (14.1 ±3.9)	39 (31.9±6.8)	3 (2.13±1.2)	9 (28.9±12.8)
Southern Appalachian	76 (62.7±8.3)	41 (48.2±9.4)	32 (25.3±7.7)	19 (28.1 ±7.0)	1 (0.16±0.14)	2 (5±3.1)	11 (11.8±5.4)	16 (18.7±5.2)
Northern Plains	13 (7.8±5.1)	7 (4.2±1.6)	18 (16.5±5.4)	21 (35.4±9.3)	–	–	34 (75.8±7.6)	43 (60.3±9.2)
Southern Plains	38 (44.3±9.3)	12 (18.7±10.5)	50 (30.7±9.1)	28 (25.4±11)	3 (2.1 ±1.7)	6 (3.2±2.1)	37 (22.9±7.2)	39 (52.7±15)
Temperate Plains	28 (16±5.4)	25 (14.6±5.8)	76 (35.4±7.3)	50 (28.7±6.5)	2 (10.7±7.5)	2 (0.15±0.1)	32 (37.7±7.6)	60 (56.5±8.8)
Upper Midwest	93 (58.2±5.4)	42 (33.1 ±7.7)	20 (11.2±3.8)	30 (19.8±5.5)	15 (17.5±3.9)	27 (31.7±9.1)	17 (13.1 ±3.7)	32 (15.4±3.6)
Western Mountains	107 (58.7±8.4)	74 (50.6±12.4)	27 (18.4±6.9)	34 (9.3±2.7)	11 (12.2±4.7)	11 (28.7±15.4)	7 (10.6±6.3)	31 (11.3±3.8)
Xeric	38 (37.5±7.1)	20 (30.4±9.3)	36 (52.5±7.9)	41 (22.5±5.4)	2 (0.29±0.18)	4 (3.2±1.7)	10 (9.7±2.8)	27 (43.9±10.2)
*National*	477 (45.7±2.7)	274 (27.7±3)	307 (20.8±2.2)	259 (20.6±2.4)	54 (10.0±1.7)	101 (16.4±3.2)	190 (23.5±2.3)	316 (35.4±3.6)

**Table 2.** Changes in true color and TP between the 2007 and 2012 NLA surveys for the 401 resampled lakes as well as information on lake size and watershed attributes. Number of lakes that changed lake class or remained in the same class are also provided.

Number of lakes		Blue to blue	Blue to brown	Blue to green	Blue to murky	Brown to blue	Brown to brown	Brown to murky	Green to blue	Green to brown	Green to green	Green to murky	Murky to brown	Murky to green	Murky to murky
		124	26	43	11	3	11	6	3	6	69	33	2	8	56
Change in color (PCU)	Min	−13	2	−8	6	−16	−2	−97	−1	5	−20	3	−2	−19	−121
	Mean	3.79	18.85	3.16	19.36	−11.33	32.91	−10.5	2.67	15.83	2.63	15.76	9.5	−10.13	6.3
	Median	4	17.5	5	15	−15	11	1.5	1	15	4	12	9.5	−9.5	5
	Max	16	38	14	44	−3	126	52	8	35	18	46	21	−2	174
Change in TP (*μ*g L^−1^)	Min	−10	−15	10	11	4	2	6	−47	−343.1	−360	−154	−11	−286	−2364
	Mean	7.52	7.31	35.33	47.18	9	5.27	33.83	−33.67	−74.63	2.81	35.03	−6.5	−29	−60.66
	Median	8	9	29	27	11	4	25	−33	−23.5	13	36	−6.5	−4.5	5.5
	Max	20	21	111	166	12	16	104	−21	−11	262	534	−2	92	780
Lake area (ha)	Min	6.21	7.76	8.21	13.84	72.28	12.02	4.72	50.17	14.49	10.59	11.75	42.06	20.67	5.01
	Mean	2997.53	372.84	939.98	107.04	127.9	87.65	94.97	2973.92	221.61	897.48	1688.41	72.76	1219.56	3729.29
	Median	90.04	82.11	195.8	84.03	127.9	43.16	49.5	88.5	112.27	170.24	125.94	72.76	29.23	48.33
	Max	125,497.42	4212.86	6559.39	533.12	183.52	282.88	382.94	8783.07	731.02	10,363.26	19,994.28	103.45	9530.15	167,489.61
% agriculture in watershed	Min	0	0	0	0.21	0.29	0	0	2.39	0.05	0	0	0	1.25	0
	Mean	10.27	8.66	23.59	19.5	31.43	0.74	0.79	29.44	10.87	30.33	32.75	3.32	34.98	26.53
	Median	2.38	1.84	12.92	12.24	31.43	0.04	0.38	28.63	5.55	21.68	30.35	3.32	39.1	19.38
	Max	64.62	70.08	74.41	67.6	62.57	4.4	3.4	57.31	27.4	87.97	82.8	6.63	62.86	81.78
% developed in watershed	Min	0	0	0.45	0.08	0	0	0	6.81	2.27	0	0	2.39	0	0
	Mean	12.14	6.84	7.46	12.34	3.15	2.55	0.8	23.69	9.84	8.79	8.87	3.32	6.48	7.34
	Median	4.61	3.83	6.04	10.06	3.15	1.08	0.61	11.47	5.47	3.71	4.41	3.32	4.18	4.13
	Max	88.96	36.69	59.21	38.13	6.29	10.3	2.16	52.78	35.23	91.04	72.71	4.25	21.07	59.1

**Table 3.** Attributes of 1013 lakes from the 2012 NLA survey which could be classified by lake class (i.e., reported total phosphorus concentration and water color).

	Blue (284 lakes)					Brown (109 lakes)					Green (273 lakes)					Murky (347 lakes)				
Attribute	Min	First quartile	Median	Third quartile	Max	Min	First quartile	Median	Third quartile	Max	Min	First quartile	Median	Third quartile	Max	Min	First quartile	Median	Third quartile	Max
*Physical characteristics*																				
Latitude (decimal degrees)	27.21	38.6	41.3	44.33	48.96	30.97	40.68	42.95	45.33	48.2	29.16	37.21	40.99	44.31	48.96	26.07	36.69	41.39	45.5	48.99
Longitude (decimal degrees)	−123.28	−111.61	−89.94	−83.29	−67.7	−124.23	−94.36	−84.6	−71.81	−67.2	−124.05	−109.86	−96.84	−88.72	−70.66	−123.93	−101.58	−96.37	−87.47	−70.89
Area (ha)	1.08	12.62	40.96	151.01	125,497	1.03	11.04	22.72	82.06	4212.86	1.16	12.92	38.09	211.43	46,299.7	1.1	9.35	22.44	71.35	167,490
Elevation (masl)	1.96	212.74	322.98	1259.52	3594.97	2.15	132.13	271.75	403.14	3032.18	0	198.02	365.04	912.18	3530.52	−53.27	122.5	346.17	635.27	3105.21
Temperature, average 0–2 m (°C)	9.69	19.86	23.9	26.69	32.02	10.32	21.1	23.66	26.95	88.62	9.58	20.44	24.1	26.82	35.2	12.12	20.84	23.94	27.42	93.07
Thermocline (m)	0.25	3.55	5.4	7.45	34.25	0.25	2.25	3.34	4.57	15.33	0.25	2.23	4.25	5.66	19.4	0.12	0.73	1.73	3.15	14.33
Secchi depth (m)	0.55	2.1	3.27	4.83	27.95	0.39	1.38	1.9	2.71	5.6	0.1	0.55	0.98	1.75	16.4	0.02	0.35	0.65	1.23	28
*Chemical characteristics*																				
Dissolved oxygen, 2 m depth (mg L^−1^)	1.49	7.57	8.42	9.3	14.5	1.55	7.17	7.93	8.62	10.6	1.25	7.13	8.06	9.21	23.5	0.3	6.25	7.67	9.21	31.8
Dissolved organic carbon (mg L^−1^)	0.4	2.03	3.11	4.82	28.1	2.05	4.36	6.14	8.31	36.1	0.23	3.69	5.4	7.57	31.2	0.58	6.34	10.06	16.43	515.81
Color (PCU)	0	7.5	11	15	20	21	25	30	44	165	0	11	15	18	20	21	25	30	38	724
Total phosphorus (*μ*g P L^−1^)	4	10	16.5	24	30	5	15	20	25	30	31	45	65	124	1208	31	59	110	297.5	3636
Total nitrogen (mg L^−1^)	0.01	0.17	0.3	0.5	6.22	0.1	0.3	0.41	0.64	2.61	0.02	0.54	0.78	1.2	5.91	0.06	0.78	1.37	2.41	54
Nitrogen : phosphorus (molar ratio)	1.75	26.26	47.4	72.84	860.67	8.77	33.95	54.09	88.57	385.29	0.57	13.78	24.51	36.45	256.86	1.77	16.06	25.27	36.08	3736.61
Dissolved inorganic nitrogen (mg N L^−1^)	0	0.01	0.01	0.02	5.96	0.01	0.01	0.01	0.02	0.32	0	0.01	0.02	0.05	5.67	0	0.02	0.02	0.05	52.12
DIN : TP ratio	0	0.95	1.86	3.11	824.54	0.44	1.06	1.48	2.39	29.34	0.02	0.36	0.65	1.43	251.14	0.02	0.23	0.52	1.27	3606.79
Calcium (mg L^−1^)	0.12	3.26	13.41	28.24	287.4	0.55	2.4	4.83	17.9	55.31	1.1	15.6	29.4	46.7	594.9	0.61	8.92	22.02	44.58	324.9
Chloride (mg L^−1^)	0.04	0.82	3.88	13.68	1975.56	0.07	1	5.31	16.57	148	0.08	4.14	11.52	23.84	1996.31	0.08	3.92	12.6	31.24	18,012.7
pH	5.38	7.33	8.09	8.46	9.53	5.1	7	7.44	8.09	9.36	3.34	8.14	8.47	8.68	10.47	2.83	7.73	8.41	8.88	10.37
Acid-neutralizing capacity (*μ*g L^−1^)	15.6	244.25	879.45	2239.75	12,529	24.1	158.2	324.1	1344	21,800	−589.6	1151	2300	3321	29,112	−3361.4	772.45	2283	3980.5	203,857
*Biological characteristics*																				
Chl *a* (*μ*g L^−1^)	0	1.51	2.81	5.19	41.84	0.87	2.83	4.68	7.76	99	0.33	5.12	15.5	35.45	373.33	0.22	9.28	24.48	62.8	764.64
Cyanobacteria density (cells mL^−1^)	0	301.31	2208.41	10,304.2	516,223	0	383.67	3234.58	14,556.4	759,826	0	1776.56	17,604.6	82,923.4	2,244,776	0	4168	34,213.3	190,111	8,757,487
Microcystin concentration (*μ*g L^−1^)	0	0	0.07	0.11	14.85	0	0	0.07	0.11	3.83	0	0.06	0.1	0.17	42.17	0	0.08	0.13	0.41	66.69
Phytoplankton biomass (*μ*g dry weight L^−1^)	0.33	61	156.9	365.74	5252.88	13.38	88.31	211.61	395.73	2154.19	0.41	193.8	579.83	1626.87	17,663.3	10.44	309.01	865.29	2718.87	61,842.5
Zooplankton biomass (*μ*g dry weight L^−1^)	0.13	20.57	49.18	108.18	1579.85	0.74	21.33	54.66	119.21	578.73	0.18	40.16	88.37	256.24	3069.62	0.16	53.97	150.04	409.74	5448.9
Zooplankton : Phytoplankton biomass ratio	0	0.11	0.32	1.12	32.5	0.01	0.07	0.24	0.82	12.45	0	0.04	0.15	0.73	336.93	0	0.04	0.16	0.48	50.25
*Watershed characteristics*																				
Wateshed area (km^2^)	0.04	2.31	8.28	49.45	604,527	0.16	2.13	11.28	39.46	1015.14	0.02	2.05	16.65	95.84	656,462	0.07	1.76	11.02	54.28	222,119
% agricultural land use	0	0	0.66	17.04	79.71	0	0	1.48	8.36	77.07	0	0.48	12.92	44.39	87.97	0	0.36	18.24	50.93	97.46
% wetland cover	0	0.01	0.77	5.13	67.96	0	1.64	5.91	14.05	60.74	0	0.16	0.99	4.5	63.8	0	0.34	2.93	9.45	94.1
% urban development	0	0.02	3.53	9.36	89.67	0	0.7	3.28	7.61	57.18	0	1.15	4.1	9.07	99.42	0	1.6	4.13	7.55	88.25
% Forest cover	0	16.42	50.89	69.71	97	0	29.99	57.08	78.39	96.81	0	1.09	14.32	46.02	96.81	0	0.32	8.47	42.75	94.6
Road density (km km^−2^)	0	0.99	1.65	2.61	16.89	0	0.95	1.56	2.31	6.08	0	1.05	1.66	2.47	18.26	0	1.11	1.52	2.17	10.12

**Table 4.** Results of Dunn tests comparing differences in Chl *a* concentrations, cyanobacteria density, and zooplankton biomass among lake nutrient-color classes. NS = not significant. Boxplots are shown in Fig. 5.

Lake comparison	Chl *a* concentration	Cyanobacteria density	Rotifer biomass	Copepod biomass	Cladoceran biomass	Total zooplankton biomass
Blue–Brown	0.001	NS	NS	0.006	NS	NS
Blue–Green	<0.0001	<0.0001	<0.0001	<0.0001	NS	<0.0001
Brown–Green	<0.0001	<0.0001	0.006	NS	NS	0.0001
Blue–Murky	<0.0001	<0.0001	<0.0001	<0.0001	NS	<0.0001
Brown–Murky	<0.0001	<0.0001	<0.0001	0.0002	NS	<0.0001
Green–Murky	0.0001	0.002	0.01	0.001	NS	0.001
