# Supplementary figures and images for: Fewer blue lakes and more murky lakes across the continental U.S.: Implications for planktonic food webs
Source: Limnol Oceanogr. Author manuscript; Available in PMC 2020 Jan 15. (PMC6961962; doi:10.1002/lno.10967)

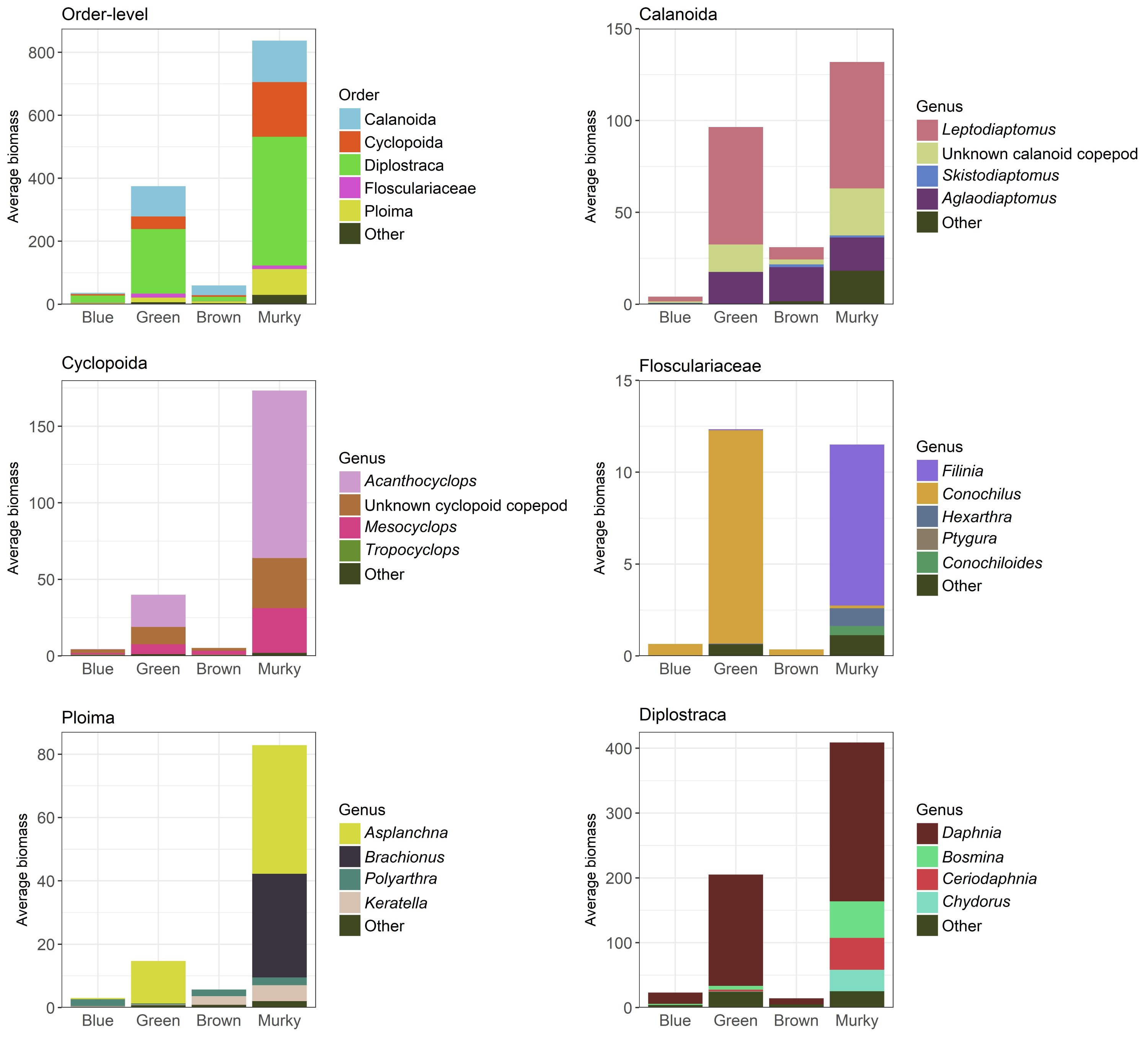

Supplement: Figure S1 [file NIHMS1052919-supplement-Figure_S1.tif]

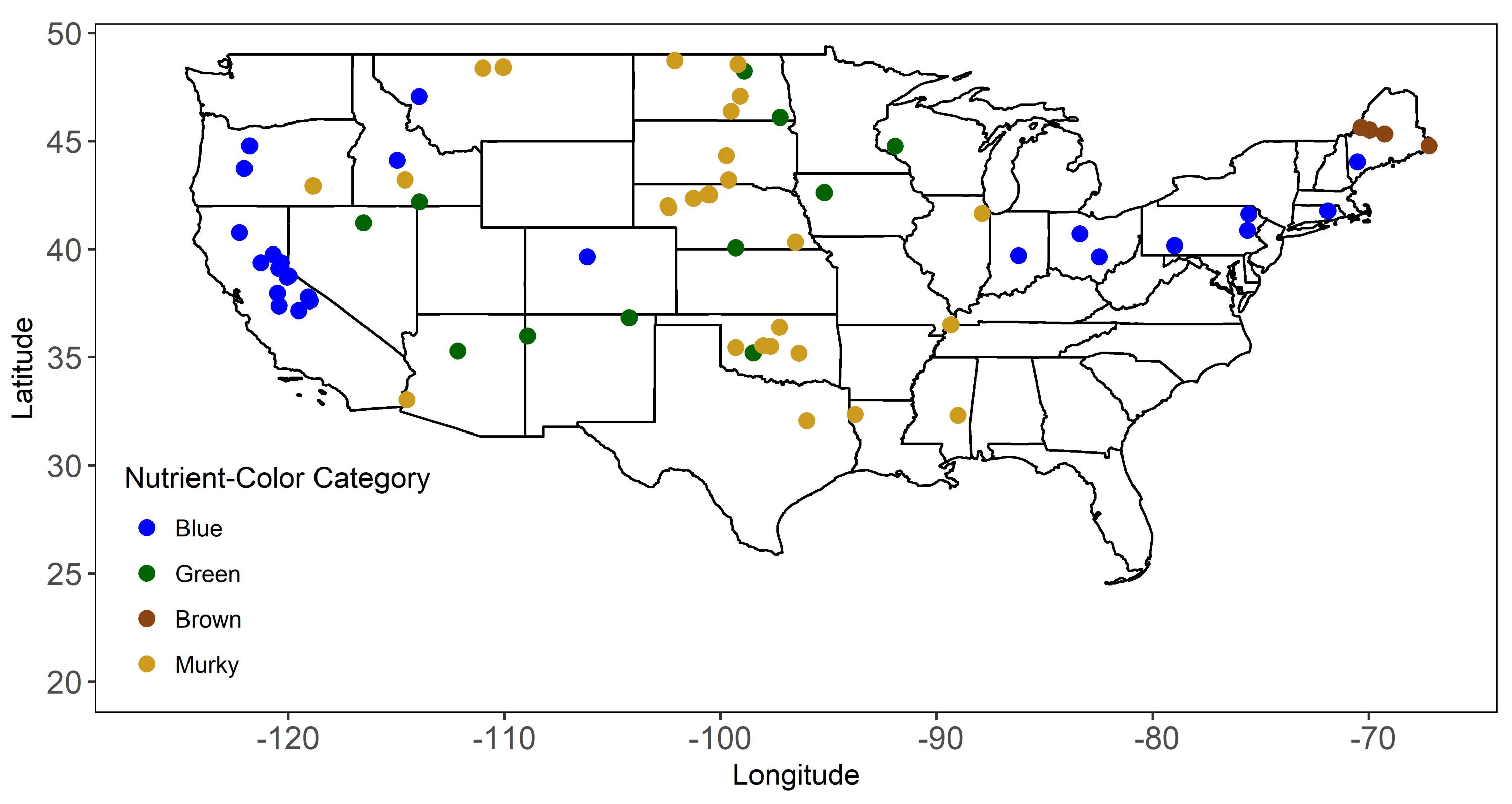

Supplement: Figure S2 [file NIHMS1052919-supplement-Figure_S2.tif]

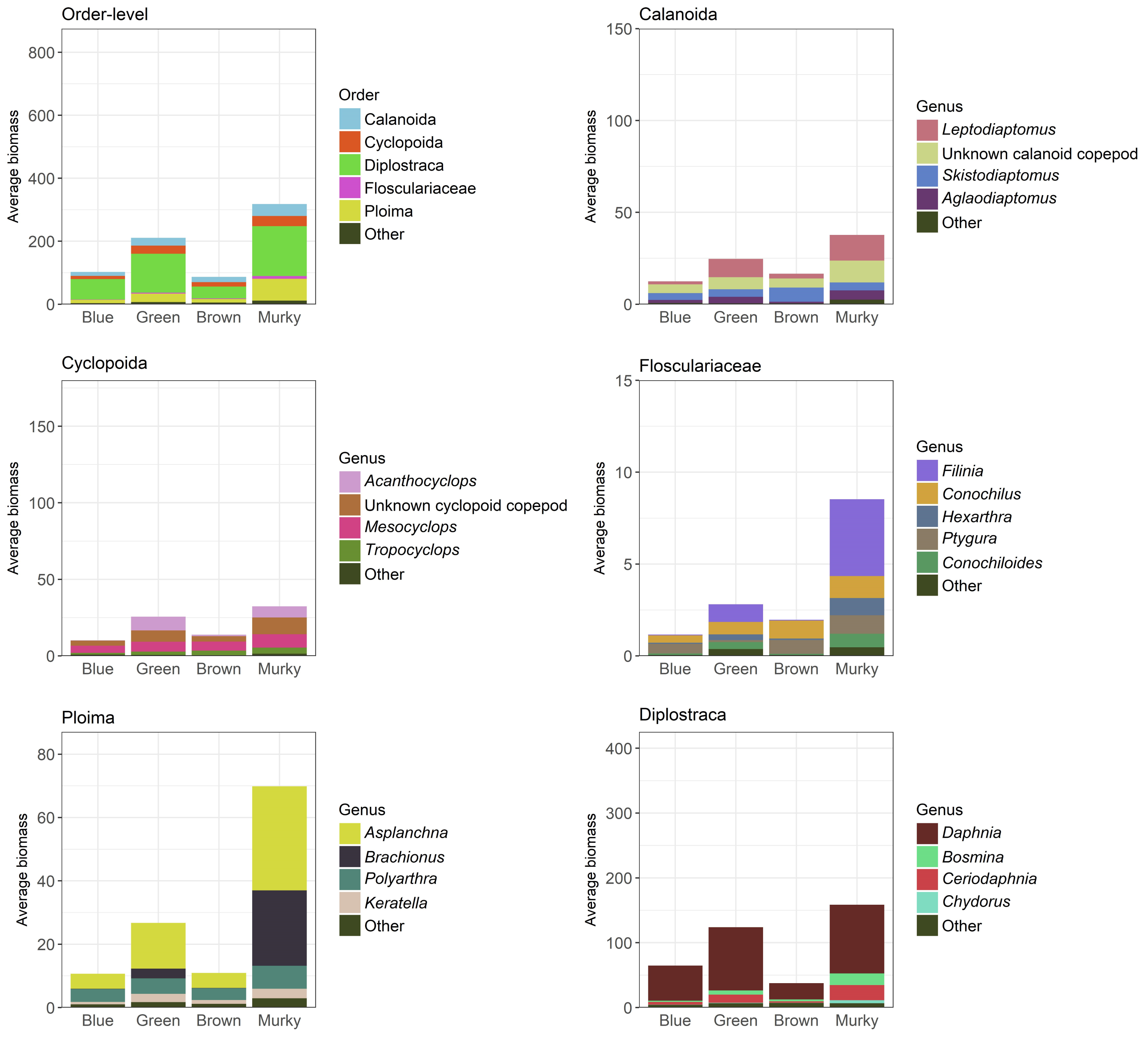

Supplement: Figure S3 [file NIHMS1052919-supplement-Figure_S3.tif]
