# Supplementary material for: Fewer blue lakes and more murky lakes across the continental U.S.: Implications for planktonic food webs
Source: Limnol Oceanogr. Author manuscript; Available in PMC 2020 Jan 15. (PMC6961962; doi:10.1002/lno.10967)
Supplement: Table S1 [file NIHMS1052919-supplement-Table_S1.docx]

**SUPPLEMENTAL TABLE**

**Supplemental Table 1.** Zooplankton biomass within each of the four lake classes, presented as µg dry weight/L for the major orders constituting > 5% of total zooplankton biomass. ‘Other’ includes the remaining identified orders: Eucopepoda, Poecilostomatoida, Collothecaceae, Podocopida, Sessilia, Veneroida, and unknown copepods and rotifers.

|  |  | **Order-level biomass** | | | | | |
| --- | --- | --- | --- | --- | --- | --- | --- |
| **Lake Class** |  | **Calanoida** | **Cyclopoida** | **Diplostraca** | **Ploima** | **Flosculariaceae** | **Other** |
| **Blue** | *Minimum* | 0 | 0 | 0 | 0 | 0 | 0 |
|  | *First quartile* | 0.2 | 0.55 | 2.69 | 0.99 | 0 | 0.77 |
|  | *Median* | 2.45 | 3.51 | 18.54 | 2.94 | 0.08 | 1.75 |
|  | *Mean* | 11.72 | 9.67 | 60.93 | 10.02 | 1.12 | 3.07 |
|  | *Third quartile* | 9.82 | 12.65 | 55.7 | 8.43 | 0.57 | 3.38 |
|  | *Maximum* | 329.49 | 130.26 | 1477.61 | 195.7 | 60.47 | 63.21 |
| **Brown** | *Minimum* | 0 | 0 | 0 | 0.03 | 0 | 0.02 |
|  | *First quartile* | 0.26 | 1.35 | 2.16 | 1.51 | 0.05 | 1.33 |
|  | *Median* | 5.86 | 5.75 | 11.38 | 4.32 | 0.18 | 2.88 |
|  | *Mean* | 17.16 | 13.56 | 36.85 | 10.75 | 1.91 | 5.38 |
|  | *Third quartile* | 19.98 | 12.69 | 48.28 | 9.4 | 1.25 | 6.38 |
|  | *Maximum* | 163.66 | 219.39 | 449.16 | 99.19 | 37.4 | 51.83 |
| **Green** | *Minimum* | 0 | 0 | 0 | 0 | 0 | 0 |
|  | *First quartile* | 0.2 | 0.91 | 2.09 | 2.02 | 0.01 | 1.4 |
|  | *Median* | 6.2 | 4.81 | 20.78 | 8.03 | 0.48 | 3.69 |
|  | *Mean* | 27.59 | 26.17 | 127.14 | 26.24 | 3.2 | 7.01 |
|  | *Third quartile* | 26.56 | 18.76 | 111.21 | 20.88 | 3.01 | 9.04 |
|  | *Maximum* | 713.12 | 754.49 | 2135.25 | 706.08 | 127.48 | 97.57 |
| **Murky** | *Minimum* | 0 | 0 | 0 | 0 | 0 | 0 |
|  | *First quartile* | 0.33 | 1.11 | 1.21 | 2.65 | 0.02 | 2.48 |
|  | *Median* | 9.1 | 7.55 | 21.03 | 11.7 | 0.54 | 6.16 |
|  | *Mean* | 45.3 | 43.77 | 178.8 | 71.22 | 8.82 | 12.88 |
|  | *Third quartile* | 51.8 | 35.06 | 128.03 | 50.22 | 3.99 | 14.11 |
|  | *Maximum* | 599.96 | 809.6 | 3254.06 | 5357.07 | 293.3 | 201.57 |
